# An Atypical Presentation of Sarcoid Myositis

**DOI:** 10.7759/cureus.31171

**Published:** 2022-11-06

**Authors:** Sydney Grubb, Logan N Rubin, Dianne Pappachristou

**Affiliations:** 1 Critical Care, Alabama College of Osteopathic Medicine, Tallahassee, USA; 2 Surgery, Alabama College of Osteopathic Medicine, Tallahassee, USA; 3 Family Medicine, Tallahassee Memorial Healthcare, Tallahassee, USA

**Keywords:** melkersson-rosenthal syndrome, autoimmune disease, extrapulmonary manifestations, granulomatosis mucositis, sarcoidosis, sarcoid myositis, atypical sarcoid

## Abstract

Sarcoidosis is a well-characterized inflammatory disease that affects multiple organ systems and can have long-term devastating outcomes if not identified and treated appropriately. The disease is most prevalent among young to middle-aged African American women. It most commonly presents with pulmonary involvement, though there are reported cases of sarcoidosis without pulmonary involvement. Pulmonary presentations can be biopsied, diagnosed, and treated with primary immunomodulation with great treatment success. Here, we present an unusual presentation of sarcoidosis as granulomatosis mucositis in the salivary gland and concurrent rare complication of sarcoid myositis in the rectus femoris in a patient with no evidence of pulmonary involvement throughout the duration of their clinical course. Further, we discuss differential diagnoses related to this patient’s presentation as well as the efficacy of treatment modalities available in the management of this disease.

## Introduction

Sarcoidosis is an inflammatory disorder characterized by granulomatous inflammation affecting multiple organ systems [1]. Commonly, the disease initially presents with chronic respiratory symptoms such as shortness of breath and a cough. Also, patients may begin to experience systemic symptoms of fever and weight loss, prompting a visit to their physician. The characteristic finding raising suspicion of sarcoidosis is bilateral pulmonary hilar adenopathy with systemic symptoms. Tissue biopsy of the adenopathy will reveal noncaseating granulomas in the absence of other systemic diseases or infections. The extrapulmonary manifestations of sarcoidosis can affect nearly every organ system in the body; skin, eye, and heart involvement are the most common of these [2]. Management of sarcoidosis largely depends on the severity of symptoms and organ involvement. Treatment primarily focuses on immunosuppression with glucocorticoids or biologic immunomodulatory medications [3]. These treatments are aimed at putting the disease into remission at which time more long-term management options can be considered such as steroids and multi-modal pain control for breakthrough symptoms. Recurrence of symptomatic sarcoidosis can be a complication of treatment causing patients to be on immunomodulatory medications long term which can themselves cause harsh organ damage due to side effects.

## Case presentation

**Figure 1:**
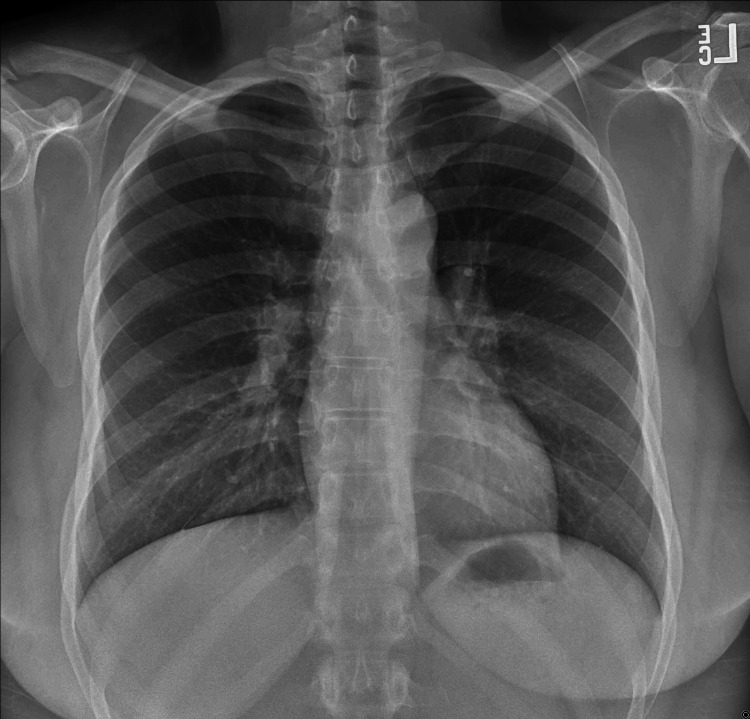
Anteroposterior (AP) chest X-ray

A 33-year-old African American female with no significant past medical history presented to her primary care physician for evaluation of a lump on her right lower jaw. The mass was enlarging and had become increasingly painful over the past two months. A physical exam revealed a one-inch nodule palpated along the right mandibular line. Initially, she was treated symptomatically with non-steroidal anti-inflammatory drugs (NSAIDs), which did not improve her symptoms. She was referred to an ENT specialist, who performed a fine needle aspiration after the patient’s symptoms persisted for three more months with a lack of response to NSAIDs. Pathology from the biopsy showed benign salivary acini with chronic inflammatory cells, suspicious for an ectopic salivary gland or neoplasm. Excision of the mass was performed by an ENT specialist and pathology results demonstrated granulomatosis mucositis. Differential diagnosis based on the biopsy and patient symptoms at that time included tuberculosis, sarcoidosis, pulse granuloma, and Melkersson-Rosenthal syndrome. Referral to rheumatology was made and the patient was diagnosed with sarcoidosis based on a pathology review and negative tuberculosis screenings. A chest X-ray was also done due to new complaints of shortness of breath but showed no pulmonary hilar adenopathy or lung abnormalities (Figure 1).

At the time of initial diagnosis, angiotensin-converting enzyme (ACE) level was 30 micrograms/L, and the sedimentation rate was 1.0 mm/hr. All other labs at this time (including complete blood count, comprehensive metabolic panel, rapid plasma reagin, and HIV) were within normal limits. The patient was started on prednisone (20mg once daily) as initial therapy. Over the next several months she developed a new lump under her right eye and an enlarging mass on her right anterior thigh. In addition to the eye lump, she had some mild blurred vision. MRI was performed and revealed bilateral lacrimal gland swelling, which was attributed to sarcoidosis. Follow-up MRI nine months later showed a decrease in lacrimal gland swelling, and the patient was no longer complaining of blurred vision. The prednisone dose was increased (60mg once daily) in an effort to manage symptoms. Her complete blood count and comprehensive metabolic panel remained within normal limits at this time. She was referred to orthopedics regarding the anterior thigh mass; there an MRI of the right thigh was ordered. This revealed an abnormal mass in the right rectus femoris muscle. A biopsy of the mass in the right rectus femoris showed non-necrotizing granulomas with fibrous stroma with chronic inflammation, leading to the diagnosis of sarcoid myositis (Figure 1) [4]. Her anti-citrullinated cyclic peptide, rheumatoid factor, and anti-nuclear antibodies were negative at this time, ruling out comorbid rheumatoid arthritis. Due to this finding of further extension of her sarcoidosis, as well as increasing severity in pain and worsening constitutional symptoms, the treatment regimen was adjusted by rheumatology to include both prednisone (taper from 60mg daily to 40mg daily) and methotrexate (10mg subcutaneous injection once weekly) for immunomodulation, as well as gabapentin (300mg three times daily) to manage pain.

Following this diagnosis of sarcoid myositis, the patient went through cyclic phases of breakthrough pain and swelling across the body, often localizing to her periorbital region. A musculoskeletal exam revealed diffuse joint swelling, but no evidence of muscle weakness, erythema, or warmth. Repeat right thigh MRI one year later revealed interval evolution of chronic rectus femoris myositis. Her episodes of swelling and symptomatic masses were attributed to flares of sarcoidosis. She additionally had an episode of facial nerve palsy and required speech therapy, which helped her return to baseline function. Her medication regimen and dosage were adjusted according to these cyclic flairs of symptoms. The patient had elected to discontinue her prednisone a few months earlier, so this was resumed at 5mg daily to aid symptom management. Hematology/oncology elected to change the patient's treatment to infliximab (5mg/kg infusion every 8 weeks) from methotrexate (25mg weekly) due to the eventual lack of symptomatic disease response to this medication. The patient showed moderate improvement in symptoms following treatment with infliximab, but this was discontinued because of an infusion reaction (headache, nausea, and urticaria). Adalimumab injections were proposed but were rejected by insurance so the patient was again trialed on methotrexate and prednisone, which has shown limited success. Through the course of her treatment with methotrexate and prednisone, her prognostic inflammatory markers rose slowly. Her maximum C-reactive protein was 18.9 mg/L and her sedimentation rate was 50 mm/hr. Creatine kinase levels rose as high as 319 IU/L. Pulmonary evaluation three years after the initial diagnosis stated there was no evidence of pulmonary sarcoidosis. Due to the failure of complete disease remission through immunomodulation, the patient has been following up with a pain management clinic in an effort to control symptoms. Her current multimodal pain control plan has consisted of ibuprofen (800mg every eight hours as needed), opioids (10mg every 12 hours as needed), duloxetine (10mg once daily), and prednisone (5mg once daily).

## Discussion

Sarcoidosis can be a challenging diagnosis to reach due to the variability in patient symptomatology. The case presented represents one of the 8% of patients that present with extrapulmonary manifestations of sarcoidosis without any detectable pulmonary involvement [5]. Her lack of pulmonary involvement is evidenced by negative chest X-rays and stable angiotensin-converting enzyme (ACE) levels. Perhaps more research should go into the presentation and frequency of these extrapulmonary manifestations, and raise clinician suspicion of sarcoidosis in patients without pulmonary symptoms at initial presentation. Additionally, this case could represent comorbid Melkersson-Rosenthal syndrome, which was considered as a differential though not formally diagnosed. This disorder is characterized by recurrent facial edema, facial muscle paralysis, and fissured tongue [6]. This disease is known to potentially precipitate other diseases, including sarcoidosis, which raises further suspicion for diagnosis. 

This case also highlights the challenges faced when treating sarcoidosis. Patients without pulmonary manifestations have clinical relapse up to 25% of the time even with adequate treatment, demonstrating the importance of close follow-up and continued medication adjustment [7]. Inappropriate management can lead to life-long pain medication requirements and potential organ dysfunction and damage. While there is a multitude of drugs used to treat sarcoidosis, there appears to be a lack of agreement in the literature on which medications to try following the failure of steroid therapy. There is a lack of trialed evidence for the superiority of immunomodulatory medications, though methotrexate is the most common second line [8]. There is an additional challenge in treating extrapulmonary sarcoidosis, and there have been few trials into the efficacy of various non-steroid drugs on different organ systems. As such, therapies are more often based on physician experience. Perhaps this case demonstrates the need for more trials into treatment for extrapulmonary sarcoidosis.

## Conclusions

Sarcoidosis is an inflammatory disease that commonly presents with pulmonary infiltrates and systemic symptoms. Many patients experience extrapulmonary involvement of the disease, such as new masses or various organ failures. We presented a case of sarcoid myositis with no pulmonary involvement. This form of sarcoidosis is challenging to diagnose because it may appear similar to benign mucositis or soft tissue swelling. The workup for diagnosing these suspected benign findings may be prolonged due to a lack of clinical suspicion for an autoimmune process, until symptoms are severe. Further, even with diagnosis, extrapulmonary manifestations are known to be much less responsive to immunomodulatory medications than classical pulmonary dominant sarcoidosis. Due to this, patients such as in this case often have continued symptoms that need long-term immunomodulation and frequent adjustments to therapy. Appropriate care for these patients requires a multidisciplinary team focused on disease regression and patient symptom management.
